# Extracorporeal membrane oxygenation support in a newborn with lower urinary tract obstruction and pulmonary hypoplasia: a case report

**DOI:** 10.1186/s13256-018-1749-1

**Published:** 2018-07-17

**Authors:** Eva Gatzweiler, Bernd Hoppe, Oliver Dewald, Christoph Berg, Andreas Müller, Heiko Reutter, Florian Kipfmueller

**Affiliations:** 1 Department of Neonatology and Pediatric Intensive Care, Children’s Hospital, University of Bonn, Adenauerallee 119, 53113 Bonn, Germany; 2 Department of Pediatric Nephrology, Children’s Hospital, University of Bonn, Adenauerallee 119, 53113 Bonn, Germany; 3 Department of Cardiac Surgery, University of Bonn, Sigmund-Freud-Straße 25, 53127 Bonn, Germany; 4 Division of Fetal Surgery, Department of Obstetrics and Prenatal Medicine, University of Bonn, Sigmund-Freud-Straße 25, 53127 Bonn, Germany; 5 Institute of Human Genetics, University of Bonn, Sigmund-Freud-Straße 25, 53127 Bonn, Germany

**Keywords:** LUTO, ECMO, Peritoneal dialysis, Hemofiltration, Pulmonary hypoplasia

## Abstract

**Background:**

Survival of neonates with intrauterine renal insufficiency and oligo- or anhydramnios correlates with the severity of secondary pulmonary hypoplasia. Early prenatal diagnosis together with repetitive amnioinfusions and modern intensive care treatment have improved the prognosis of these neonates. Extracorporeal membrane oxygenation is an established treatment option, mainly applied to neonates with pulmonary hypoplasia caused by congenital diaphragmatic hernia. However, a few case reports of extracorporeal membrane oxygenation in neonates with lower urinary tract obstruction have been published.

**Case presentation:**

We describe a case of a Caucasian male infant with prenatally diagnosed lower urinary tract obstruction and secondary pulmonary hypoplasia who was delivered spontaneously at 36 + 2 weeks of gestation. Venovenous extracorporeal membrane oxygenation was initiated on the first day of life for severe respiratory failure and consecutive hypoxemia despite treatment with inhaled nitric oxide and high-frequency oscillation. The patient was supported by extracorporeal membrane oxygenation for 10 days and extubated 6 weeks later. Hemofiltration was required on the second day of life because of renal insufficiency and was later replaced by peritoneal dialysis. The child was discharged after 4 months with nasal high-flow mild oxygen therapy and peritoneal dialysis.

**Conclusion:**

Neonatal extracorporeal membrane oxygenation support is a possible treatment option for neonates with lower urinary tract obstruction and pulmonary hypoplasia.

## Background

Fetal renal insufficiency is often associated with oligo- or anhydramnios and secondary pulmonary hypoplasia. Although multiple factors promote fetal lung growth, a sufficient amount of amniotic fluid is paramount for normal lung development. Infants with pulmonary hypoplasia present with a decreased density of the bronchial structures and pulmonary vasculature. These infants might present with respiratory failure and pulmonary hypertension. Pulmonary function is crucial concerning survival in the first week of life. Early prenatal diagnosis, amnioinfusion, placement of a feto-amniotic shunt, and modern intensive care treatment have improved the prognosis of these children [1]. Despite these therapeutic options, death caused by respiratory failure is still high. Long-term morbidity depends on the prevalence of chronic renal impairment [2].

Extracorporeal membrane oxygenation (ECMO) is an established treatment applied in neonates with pulmonary insufficiency to bridge time for pulmonary recovery or to overcome persistent pulmonary hypertension [3]. ECMO led to an increased survival rate for some indications, such as meconium aspiration syndrome. Today the most common indication for ECMO in neonates is congenital diaphragmatic hernia (CDH). However, ECMO for CDH is not unequivocally accepted among some experts, because there is no clear evidence for a survival benefit in these patients. We describe a case of a neonate with lower urinary tract obstruction (LUTO), secondary pulmonary hypoplasia, and severe respiratory failure successfully treated with application of early ECMO support.

## Case presentation

A Caucasian male neonate (birthweight 2340 g, < 10th percentile) with a prenatal diagnosis of LUTO and pulmonary hypoplasia was delivered spontaneously at 36 + 2 weeks of gestation. LUTO caused by posterior urethral valves was diagnosed by prenatal ultrasound at 20 + 5 weeks of gestation due to megacystis with a pathognomonic sonographic keyhole sign. Further, moderate oligohydramnios (amniotic fluid index of 5.1 cm with single deepest pocket of 1.5 cm), bilateral ureteral dilation, and hydronephrosis were present. Amnioinfusion was administered successfully once at 21 + 5 weeks of gestation.

Postnatal adaptation was impaired with an Apgar score of 5/6/7. At birth, the infant was found to be cyanotic with severe tachydyspnea and chest wall indrawing, and his capillary refill time was prolonged. Because of respiratory insufficiency, the boy was intubated in the first hour of life and received pressure-controlled ventilation. Pulmonary hypertension was diagnosed by echocardiography using ductus arteriosus flow pattern, position of the intraventricular septum, and tricuspid regurgitation according to the method used by Lusk *et al.* [4]. Suprasystemic pulmonary hypertension was observed, and the boy was treated with inhaled nitric oxide (20 ppm) and intravenous sildenafil (1.6 mg/kg/day administered continuously). Owing to pleural effusion of the left lung and pneumothorax of the right lung, two chest drains were placed within the first hours of life. Despite intensive high-frequency oscillation (mean airway pressure 15 cmH_2_O, fraction of inspired oxygen [FiO_2_] 1.0) and repetitive administration of surfactant, oxygenation indices according to Ortiz *et al.* [5] were above 40 after 5 hours of life. Before ECMO cannulation, partial pressure of carbon dioxide (PaCO_2_) was 53 mmHg, and partial pressure of oxygen (PaO_2_) was 34 mmHg. Thus, venovenous ECMO with a double-lumen cannula (13-French AVALON Elite Bi-Caval Dual Lumen Catheter; Maquet/Getinge, Gothenburg, Sweden) was initiated using a rotational pump (deltastream® DP3; Xenios, Aachen, Germany) and maintained for 10 days without complications. During the ECMO run, oxygen supply could be reduced and tidal volumes increased. Severity of pulmonary hypertension was assessed daily by echocardiography during ECMO and decreased to approximately one-third of systemic pressure within 24 hours on ECMO support. The patient was extubated after 6 weeks of ventilation and supported with continuous positive airway pressure and later with nasal high-flow and mild oxygen therapy.

Owing to renal insufficiency with anuria, continuous venovenous hemofiltration (CVVH) was started on the second day of life via the ECMO circuit. After ECMO decannulation, hemofiltration was first continued via a Shaldon catheter until a Tenckhoff catheter was implanted 1 week later. Two weeks after ECMO decannulation and hemodynamic stabilization, peritoneal dialysis was effective and CVVH could be discontinued. After operative revision of the Tenckhoff catheter, the patient developed a sepsis with peritonitis caused by *Enterococcus faecium*, which was treated successfully with antibiotics.

After 4 months, the patient could be discharged from hospital care with peritoneal dialysis and nasal high-flow oxygen (flow 11 L/minute, FiO_2_ 0.21–0.3). At 1-year follow-up, the child was supported by nasal oxygen supply with 0.25 L/minute and was in preparation for kidney transplant with a body weight of 7.5 kg.

## Discussion and conclusions

Many studies have been conducted to assess the degree of renal insufficiency in fetuses with LUTO, either to identify candidates for prenatal interventions or to predict postnatal outcome [6]. In a systematic review by Morris *et al.* [7], volume measurements of amniotic fluid and fetal renal cortical appearance demonstrated the highest predictive values for postnatal renal function. In contrast, the prenatal assessment for pulmonary hypoplasia, and especially the prediction of postnatal respiratory failure, in fetuses with prenatal renal failure and secondary renal oligohydramnios (ROH) is less accurate [2].

Oligohydramnios caused by fetal renal failure is associated with impaired maturation of the lungs and secondary pulmonary hypoplasia. The severity of respiratory failure during initial postnatal transition is the main determinant for survival, whereas long-term morbidity mainly depends on the need for dialysis or kidney transplant [2]. Persistent pulmonary hypertension contributes to the severity of respiratory failure and is frequently present in children with pulmonary hypoplasia, regardless of the underlying etiology [8].

LUTO is associated with persistent oligo- or anhydramnios leading to life-threatening pulmonary hypoplasia [9]. The overall mortality of patients with ROH ranges between 30% and 40% in several studies, although direct comparison of these studies should be made cautiously owing to the heterogeneous etiologies of ROH. Mehler *et al.* [10] described 36 newborns with ROH caused by obstructive uropathy (*n* = 19), polycystic kidney disease (*n* = 6), renal agenesis/dysplasia (*n* = 10), and bilateral renal vein thrombosis (*n* = 1). In their report, 47% of the 15 nonsurvivors died of respiratory failure. Similar results were reported by Klassen *et al*. [2] with an overall mortality of 30% (7 of 23) in newborns with ROH (congenital anomalies of the kidney and urinary tract [*n* = 16], polycystic kidney disease [*n* = 4], renal tubular dysgenesis [*n* = 3]), and in 4 (57%) of 7, mortality was attributable to early respiratory failure.

In patients with severe neonatal respiratory failure, ECMO is a standard treatment option. The introduction of ECMO support has improved the survival of newborns with respiratory failure of various underlying diseases. The survival rate of infants with ECMO and CDH is approximately 60% in most centers. Because of the similarity of the observed histological changes in pulmonary development in infants with LUTO and CDH, we believe that the survival rate of ECMO applied for infants with LUTO and severe respiratory failure might be in the same range as for infants with CDH. In a study done in Chile, Kattan *et al.* [11] analyzed the outcome of neonates before and after the introduction of ECMO and observed a sharp increase in survival, especially in the CDH-group. To avoid unnecessary ECMO support in the most severely affected infants, some centers use selection criteria for ECMO in CDH patients (for example, at least one stable period with PaO_2_ > 60 mmHg or PaCO_2_ < 60 mmHg). These selection criteria, which were also proposed by Hoffman *et al.* [12], might also be reasonable for patients with LUTO and severe respiratory failure to exclude infants with extreme lung hypoplasia. Our patient was stabilized with a PaCO_2_ < 60 mmHg before ECMO cannulation.

Regarding patients with congenital renal failure and pulmonary hypoplasia, a few case reports describe the use of ECMO. Caesar *et al*. [13] reported cases of four newborns with ROH, postnatal renal insufficiency, and postnatal carbon dioxide retention with the need for mechanical ventilation. In all four newborns, ECMO was used because of respiratory failure after failure of mechanical ventilation. Three of the four patients survived to hospital discharge (follow-up from 7 months to 5 years). The nonsurvivor died of bilateral renal dysplasia with progressive azotemia and the decision not to install dialysis.

Neonatal pulmonary ECMO has the highest survival rate compared with respiratory pediatric or adult ECMO, with a survival rate of 73% to discharge or transfer [14]. General inclusion criteria for ECMO are a gestational age > 34 weeks, a body weight > 2 kg, and the existence of a potentially reversible respiratory failure. Neonates with intraventricular hemorrhage greater than first degree are usually excluded. The major risks of ECMO therapy are bleeding complications and mechanical problems. According to Paden *et al.,* the incidence of intraventricular hemorrhage was 7% in neonatal respiratory ECMO, and cannula problems occurred in 11.6% of the cases [15]. Nevertheless, the high survival rates and favorable neurodevelopmental outcome justifies use of ECMO for neonatal respiratory failure. Authors of a Cochrane meta-analysis also concluded that use of ECMO in neonates with severe but possibly reversible respiratory failure results in improved survival [16].

The neurological long-term outcome of children with congenital renal failure has not yet been studied prospectively, but it seems to be appropriate for age in most patients [2]. In a prospective follow-up study by van den Hondel *et al.* [17], 24.3% of the ECMO survivors had hearing loss, but they showed normal intelligence scores and language skills at the age of 5 years. Several long-term outcome studies on children with congenital renal failure have been done with a focus on renal outcome and general survival, not taking into account the possibility of ECMO support. When counseling parents of fetuses with intrauterine renal failure, it should be mentioned that these infants, especially when ECMO support is initiated, might face numerous long-term complications due to domestic peritoneal dialysis, pulmonary hypertension, chronic respiratory disease, impaired hearing, or developmental delay.

The use of ECMO might be considered for newborns with ROH and high risk of respiratory failure. In our experience, in infants with severe pulmonary hypoplasia, survival is extremely unlikely if PaCO_2_ does not decrease below 80 mmHg in the first 6 hours of life despite optimal conventional treatment. Additionally, newborns with consistent profound hypoxia, such as the inability to achieve PaO_2_ > 35 mmHg or oxygenation index < 50 by using lung-protective ventilation (that is, peak inspiratory pressure ≤ 28 cmH_2_O), will most likely die. Therefore, we would consider this as a contraindication to ECMO support. However, additional factors such as birthweight (< 1800 g), gestational age (< 32 weeks), and the parents’ request must be considered [18]. The aforementioned criteria reflect our personal experience and can only serve as guidance in decision-making. In our center, we might provide ECMO support even in the most severe cases if the parents insist on use of ECMO after thorough counseling, although this situation did not occur in our department in the last decade. However, neonates with mainly oxygenation problems and/or suprasystemic pulmonary hypertension but with preserved ventilation (PaCO_2_ < 60 mmHg) and sufficient lung volume (> 3–4 ml/kg body weight), ECMO support should not be withheld. Thus, prenatal transfer to an ECMO center should be discussed for fetuses with congenital renal dysfunction and expected early respiratory failure.

## Abbreviations

CDH: Congenital diaphragmatic hernia
CVVH: Continuous venovenous hemofiltration
ECMO: Extracorporeal membrane oxygenation
FiO_2_: Fraction of inspired oxygen
PaCO_2_: Partial pressure of carbon dioxide
PaO_2_: Partial pressure of oxygen
LUTO: Lower urinary tract obstruction
ROH: Renal oligohydramnios
